# Stress evaluation in one-piece and bone- or tissue-level two-piece zirconia dental implants with ceramic or titanium abutment screws: a finite element analysis

**DOI:** 10.1186/s12903-026-08192-y

**Published:** 2026-04-23

**Authors:** Abdulaziz Gul, Emil Bureau, Jonas P Becktor, Per Vult von Steyern, Evaggelia Papia

**Affiliations:** 1 Department of Materials Science and Technology, Division 2, Faculty of Odontology, Malmö University, Malmö, SE-205 06 Sweden; 2 Department of Oral and Maxillofacial Surgery and Oral Medicine, Division 2, Faculty of Odontology, Malmö University, Malmö, SE-205 06 Sweden; 3 Department of Oral and Maxillofacial Surgery, Faculty of Dental Medicine, Umm Al-Qura University, Makkah, 21955 Saudi Arabia; 4 Det Tekniske Bureau, Copenhagen, Danmark; 5 The Nordic Institute of Dental Materials, NIOM, Oslo, 0855 Norway

**Keywords:** Bone-level, Ceramics, Dental implants, Finite element analysis, Implant design, One-piece implant, Screw material, Stress analysis, Tissue-level, Zirconia

## Abstract

**Background:**

Various designs of dental implants made of zirconium dioxide (zirconia) as an alternative to titanium implants are available as one-piece implant (OPI) or as bone-level or tissue-level two-piece implants (TPI) with either ceramic or titanium abutment screw types of implant-abutment connections. This study aimed to identify and quantify stress concentrations and levels across different zirconia implant designs under a quasi-static external load.

**Methods:**

The study used a finite element analysis (FEA) simulation model, including five zirconia implant design models of OPI and bone-level and tissue-level TPI zirconia implants with either ceramic or titanium abutment screw, to calculate and locate maximum principal (σ_1_) and minimum principal (σ_3_) and equivalent (von Mises) stresses under 400N of quasi-static loading at a 30-degree angle within ISO14801:2016 geometry and boundary conditions. The FEA assumed linear elastic and isotropic material properties.

**Results:**

OPI showed high stress concentrations at the implant body’s external thread above the specimen holder. TPIs showed similar maximum principal stress (σ_1_) areas in their implant body, while minimum principal stress (σ_3_) occurred in the implant–abutment interface at the abutment neck, and the corresponding area in the implant platform. The TPIs with ceramic screws exhibited the highest stresses at the screw–abutment interface.

**Conclusions:**

Within the limitations of this study, under a static oblique load applied within the ISO 14801:2016 geometry condition, stress distributions differ between OPI and TPI. TPIs show lower stress within the implant body but higher stress transfer to the specimen holder compared with OPI. Tissue-level TPIs exhibit lower stress than bone-level designs, whereas stresses in the abutment and screw depend on screw design and material. TPIs with ceramic screws exhibit higher stress levels compared to titanium screw.

## Background

Recent advances in the field of dental implants include the reintroduction of ceramics, such as zirconium dioxide (ZrO2, zirconia), owing to their biocompatibility and mechanical properties [1–5]. Yttria-stabilized tetragonal zirconia polycrystal (Y-TZP) is notable for its high fracture toughness and resistance to crack propagation. Stress-induced phase transformation, or transformation-toughening, causes the tetragonal phase to transform to monoclinic near a crack tip under stress, increasing volume and generating a force that prevents crack propagation [1, 3, 6].

Generally, two main designs of zirconia dental implants are available in the market: one-piece implant (OPI) and two-piece implant (TPI). In the OPI design, the implant and the abutment are integrated into one monolithic unit. In previous finite element analysis (FEA) studies, zirconia-based OPIs showed the maximum stress levels in the implant body first and second threads at the neck region [7]. Moreover, when subjected to axial and oblique loading conditions, OPIs were reported to transfer less stress to the peri-implant bone structure than comparable TPIs [8].

A zirconia-based TPI design consists of two separate parts (implant body and abutment) assembled by either a screw, adhesive cementation, or a combination of both, which are provided as bone-level (BL) implant or tissue-level (TL) implant. Compared to BL implants, TL ones have a smooth transmucosal extra collar above the rough and threaded part of the implant body, allowing for transmucosal soft tissue attachment. Since zirconia components exhibit material properties that differ from titanium alloys, which are traditionally used to manufacture dental implants, they are expected to respond differently under cyclic or static external loading. Furthermore, zirconia is a brittle material with a high elastic modulus, and installing a zirconia abutment on a zirconia implant body can induce significant stresses under loading, which may, in turn, lead to crack initiation and fracture [9]. To achieve a stable implant–abutment connection, and to accommodate patient preferences such as metal-free restorations, zirconia-based TPIs are sometimes combined with screws and abutments manufactured from other materials, such as titanium, polyether ether ketone (PEEK), or crystalline polyether ketone ketone (PEKK) [10–13]. The reported technical complications with zirconia-based TPI are abutment and implant body fractures [14–19]. These complications were attributed to impurities incorporated in the material during the manufacturing process and to stress concentration in the implant design [12, 17–19]. Although zirconia possesses stress-induced transformation-toughening properties, excessive stress concentrations may lead to crack formation, propagation, and finally fracture [20–22]. Thus, the stress levels and concentrations in different designs of zirconia dental implants and prosthetic complexes should be carefully considered.

Since the zirconia-based BL and TL TPI design with ceramic and titanium abutment screws were more recently developed compared to the OPI design, research evaluating the stress levels and the regions of high stress concentrations in different designs and material combinations is limited. Therefore, it is important to examine how stresses from applied loads are distributed depending on design and material choice. This knowledge would benefit the future design development for manufacturers and the design selection process for clinicians to provide patients with the best possible care.

FEA is a numerical approach used to simulate controlled mechanical conditions, in which external loads may be applied axially or obliquely to analyse stresses in different implant–abutment design configurations. The geometry and loading conditions defined in ISO 14801:2016 enable direct comparison of mechanical performance and stress distribution between different models. In addition, this standardized loading configuration facilitates comparison between numerical simulations and analogous physical experiments [23, 24].

The aim was to identify and quantify stress concentrations and levels in five different zirconia implant designs (i.e., OPI and BL or TL TPI with a ceramic or titanium abutment screw).

Based on the aim, the research question was:

Where are concentrations of high stress (maximum principal (σ_1_), minimum principal (σ_3_) and equivalent von-Mises) located in five different zirconia implant designs—OPI and BL or TL TPI with a ceramic or titanium abutment screw—when subjected to quasi-static external loading conditions using ISO 14801:2016-defined geometry and boundary conditions?

## Methods

### FEA model and ISO14801:2016 geometry and loading setup

**Fig. 1:**
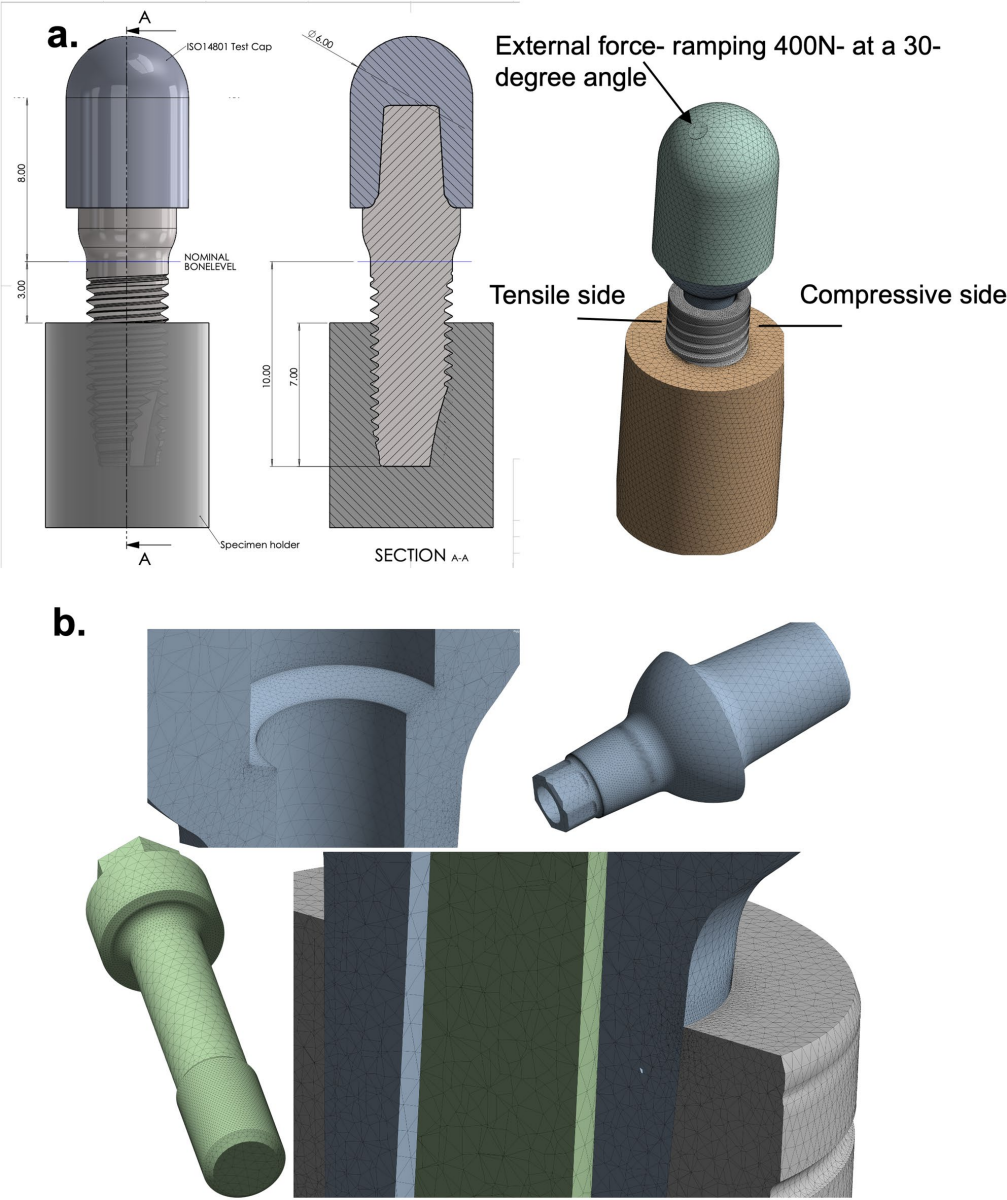
Simulation setup in accordance with the ISO 14801:2016 requirements. **a** The images show the CAD assembly and the setup of the simulation and test model following the ISO 14801:2016 requirements, i.e., external load at a 30-degree angle, implant body position at a 3 mm offset to simulate boundary conditions of bone loss, embedding material with a elastic modulus of 3 GPa, and a loading cap designed with an 11 mm distance from the centre of the loading cap to the level of the specimen holder. **b** The Finite Element Analysis Mesh

**Fig. 2:**
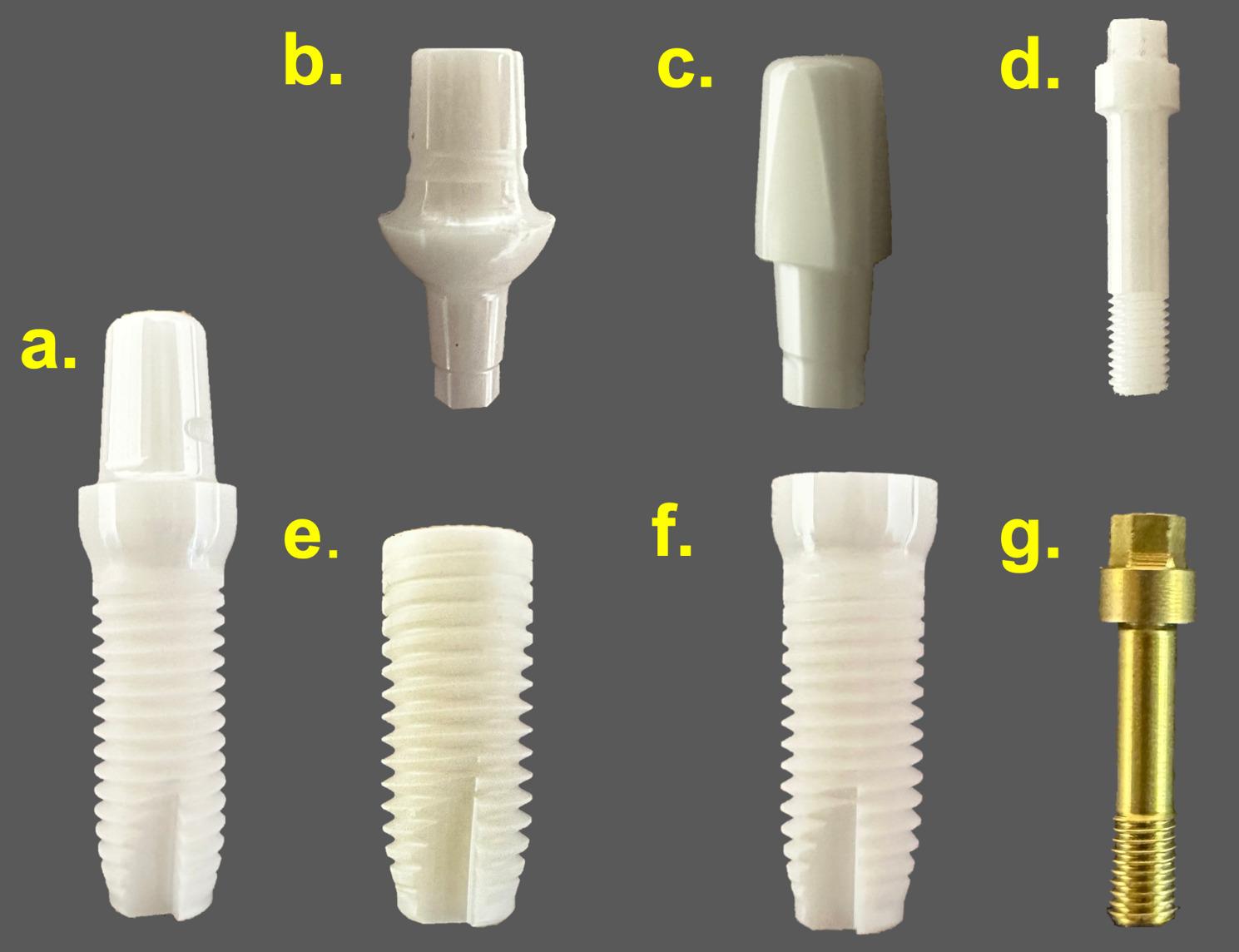
The implant-abutment complex design components used in the study and their product names. **a** one-piece implant (product Z5M, Z-Systems, Switzerland); **b** bone-level two-piece abutment (BL-A0025, Z-Systems); **c** tissue-level two-piece abutment (TL-A0055, Z-Systems); **d** ceramic abutment screw (BL-OSC-H, Z-Systems); **e** bone-level two-piece implant body (Z5BL, Z-Systems); **f** tissue-level two-piece implant body (Z5TL, Z-Systems); and (**g**) titanium abutment screw (BL-OST-H, Z-Systems)

Five separate FEA models were built (using Ansys Mechanical 2025 R1) to simulate, analyse, and compare stresses under quasi-static loading conditions. The models included five different design models of zirconia dental implants made of Y-TZP (Z-Systems, Switzerland). Model 1 is an OPI, and the remaining models are TPIs: Model 2, a bone-level two-piece with a titanium screw (BLTS); Model 3, a bone-level two-piece with a ceramic screw (BLCS); Model 4, a tissue-level two-piece with a titanium screw (TLTS); and Model 5, a tissue-level two-piece with a ceramic screw (TLCS). All implant body models have a 4.0 mm diameter and a 10 mm length. A STEP files for each component of the implant-abutment complex of the five models were supplied by the manufacturer (Z-Systems, Switzerland). The three-dimensional computer-aided design (CAD) models were prepared for FEA (using SolidWorks 2024). Additional models and component details are provided in Tables 1 and 2; Figs. 1 and 2.

**Table 1:** Implant-abutment models geometry and specifications

Main designs	One-piece implant	Two-piece implant, titanium screw		Two-piece implant, ceramic screw	
Manufacturer	Z-Systems				
Product	Z5m	Z5-BL	Z5-TL	Z5-BL	Z5-TL
Intraosseous diameter (mm)	4	4	4	4	4
Intraosseous length (mm)	10	10	10	10	10
Shoulder height (mm)	≈ 2.9	n/a	≈ 2.5	n/a	≈ 2.5
Abutment length (mm)	5	≈ 7.5	≈ 5.5	≈ 7.5	≈ 5.5
Implant–abutment connection	One-piece	Two-piece with titanium screw M1.4x0.2 14 Ncm pretension		Two-piece with teramic screw M1.4x0.2 14 Ncm pretension	
		Internal conical connection			

*Abbreviations*: *BL* bone-level, *TL* tissue-level

**Table 2:** Design models used in the study

Zirconia dental implant study models				
**Model 1**: One-piece implant (OPI)	Two-piece implant (TPI)			
	Bone-level (BL)		Tissue level (TL)	
	**Model 2**: Bone-level two-piece implant with titanium screw (BLTS)	**Model 3**: Bone-level two-piece implant with ceramic screw (BLCS)	**Model 4**: Tissue-level two-piece implant with titanium screw (TLTS)	**Model 5**: Tissue-level two-piece implant with ceramic screw (TLCS)

The models were assembled, and a hemispherical loading cap was designed in accordance with ISO 14801:2016 requirements. The height of the loading cap for each design model was compensated based on simple laboratory experiments to measure the height of the compressed assembled models after applying the abutment screw tensioning moment (Mt = 14 Ncm) required by the manufacturer. (Fig. 1a). The loading cap was designed with a surface that enabled the precise application of an external load at 30-degree in the FEA model (Fig. 1a and b) [23]. The implant models were inserted in specimen holders according to the ISO 14801:2016; the specimen holder’s elastic modulus was 3 GPa, and the embedding level was placed 3 mm below the implants’ nominal bone level, resembling the boundary conditions of an implant body with bone loss. In the FEA, the outer cylindrical surface and bottom of the specimen holders were fixed to mimic laboratory testing conditions (see Fig. 1a).

The applied load was quasi-static, ramped 0–400 N. The term quasi-static refers to the load not being a dynamic, cyclic, or immediately applied (stepped) load, but instead ramped up slowly enough to ignore dynamic effects such as inertia. Ramping of the load helps to resolve the micro-movement between parts in the frictional interfaces and aids simulation convergence. The ramping force Fext(t) is defined by the following Eq. (1):

1 $$\:{F}_{ext}\left(t\right)=\left\{\begin{array}{c}0,\:\:t\:<0\\\:\left(t-{t}_{0}\right)*{F}_{full},\:\:t\wedge\:\:{t}_{0}\ge\:0,\end{array}\right.$$

where $\:t$ is the current simulation time step, *t*_*0*_ denotes the load step starting time, and *F*_*full*_ is the final full load to be applied. In the present simulation, the screw pretension was applied during the time step *t = 0–1 s* and the subsequent external load *F*_*ext*_*(t)* was applied in the time step *t = 1–2 s*, reaching its full value of *F*_*full*_ *= 400 N at t = 2s.* The external load (*F*_*est*_*)* was applied to the loading cap at an angle of 30 degrees from the vertical plane as described by ISO 14801:2016.

### Screw pretention

The CAD models were assembled in an uncompressed state and imported to Ansys, where the screw tensioning was explicitly simulated, and the resulting compression and seating of the parts were resolved, similarly to how it was done in Choi et al. [25]. With a tensioning moment of (*Mt* = 14 Ncm), the resulting pretension force *Fpt* in the screw was different between the design with the titanium abutment screw (*Fpt*, Titanium = 304 N) and the one using the ceramic abutment screw (*Fpt*, Zirconia = 340 N) due to the screw-abutment interface design being different. The estimation of pretension force (*Fpt*) from the tensioning moment (Mt) depends on the screw-abutment interface geometry, thread type, and friction coefficient(s) and was calculated as described by Lang et al. [26].

### Friction estimate

Since the friction coefficient depends on both material properties and manufactured surfaces,it was estimated for the specific assembly utilized in the present study. For simplicity, allinterfaces were assigned the same friction coefficient, µ= 0.25, and no differentiation wasmade between sliding and static friction. The friction coefficient was estimated using theexperimentally measured height difference (travel length) between the uncompressed andcompressed assemblies of the physical components and comparing it with the simulatedtravel length during the pretension step. This process was iterated until satisfactory agreementwas achieved between experimental and simulated results across all models.

### Contact interfaces

The FEA models used a mix of bonded and frictional interfaces between components, see Table 3. Frictional interfaces play a significant role during pretension and external loading. In the pretension step, they allow component interaction and sliding, thereby stabilizing the deformed, pre-stressed condition of the implant-abutment complex. During external loading, these interfaces permit relative sliding of parts, accommodating stresses and deformations. Small levels of allowable penetration were permitted in the frictional interfaces and controlled by the simulation parameter normal stiffness factor (FKN), see Table 3. This was done to compensate for the artificially high stiffness resulting from the absence of a damage model and to aid in contact stabilization. The penetration levels were carefully checked to ensure negligible effect relative to both local and global displacements (the largest observed level of penetration during simulation across all five models was 2.17 × 10⁻⁴ mm). 

In addition to reduced contact stiffness, other contact parameters were modified (see Table 3). The internal implant threads and the external abutment screw threads were removed and replaced by a cylindrical geometry with a diameter matching the mean pitch diameter of the thread. The threaded contact was modelled using Ansys’ thread algorithm, allowing calculation of stresses in the threads at a much lower computational cost than explicit modelling of the geometry, and without simplifying the interface to use a bonded contact. In selected contact pairs, the Ansys’ *adjust to touch* option was used, see Table 3, to eliminate initial gaps or penetrations between contacting elements. This both reduces the effect of potential inaccuracies of the CAD models and the generated mesh. Thereby improving contact stabilization and reducing sensitivity to minor CAD or mesh inaccuracies. The effect of these settings was checked for all relevant contact pairs. 

**Table 3:** Contact pairs used in the analysis

Contact Pair	Contact type	Interface description	Contact modifications	Notes
Implant to specimen holder	Bonded	The external threads of the implant body are bonded to the specimen holder	-	The internal part of the CAD model of the specimen holder is created to exactly match the external geometry of the implant.
Implant to screw	Frictional, *µ* = 0.25	Threaded connection between the abutment screw and the implant body	Bolt thread, adjust to touch. (FKN = 1)	Internal threads were removed from the CAD model and replaced by cylindrical geometry with a diameter matching the mean pitch diameter of the thread.
Implant to abutment, hex part	Frictional, *µ* = 0.25	Possible contact between the abutment hex and the implant body	Reduced contact stiffness (FKN = 0.15)	The contact pair is open during the screw pre-tension step, but comes in contact on one side during the external loading step.
Implant to abutment, conical part	Frictional. *µ* = 0.25	Conical connection between implant body and abutment	Reduced contact stiffness (FKN = 0.15)	-
Abutment to screw	Frictional, *µ* = 0.25	Contact between abutment and screw abutment head	Adjust to touch, Reduced contact stiffness (FKN = 0.15)	For the titanium screw, the interface is the flat surface under the screw head, and for the zirconia screw geometry is a line contact.
Abutment to loading cap	Bonded	Contact between the abutment and the loading cap	-	

*Abbreviations*: *CAD* Computer-aided design, *FKN* normal stiffness factor *µ* friction coefficient

### FEA mesh and convergence 

The FEA mesh for each model was defined with 800,000–1,200,000 elements, mainly tetrahedral, with quadratic shape functions. However, the mesh is highly refined in regions of high stress concentration (Figure 1b), and great care was taken to verify that the mesh density was sufficient to achieve the correct mechanical behaviour at the frictional interfaces. All critical results were checked for convergence with respect to mesh density.

### Material specifications

All materials were assumed to be linear elastic and isotropic. Table 4 lists the material characteristics used for all design components [24]. 

**Table 4:** Material characteristics of the implant body, abutment, abutment screw, loading cap, and specimen holder

Component	Material and content	Young’s modulus (GPa)	Poisson’s ratio	Tensile yield strength (MPa)	Ultimate tensile strength (MPa)	Ultimate compressive strength (MPa)
Implant body, abutment (TL, BL), and abutment screw^†^	Zirconia Y-TZP (HIP) ZrO2/Y2O3/Al2O3 95/5/0.25 wt%	210	0.31	-	1200	2000
Titanium screw^‡^[24]	Titanium Grade 5 Ti6AL/4V ELI	114	0.33	940	1054	-
Specimen holder	Acrylic plastic (PMMA)	3	0.4	-	-	-
ISO loading cap	Structural steel	200	0.3	-	-	-

^**†**^, Characteristics provided by the manufacturer (Z-Systems)

^**‡**^, Characteristics retrieved from de la Rosa et al. [24]

*Abbreviations*: *Y-TZP* Yttria-stabilized tetragonal zirconia polycrystal, *HIP* heat isostatic pressure, *ELI* extra low interstitial, *TL* tissue level, *BL* bone level

### Post processing

The stress distribution and levels in the FEA models were analysed by evaluating the equivalent (von-Mises) stress and maximum principal (σ_1_) and minimum principal (σ_3_), each used when appropriate. The equivalent (von Mises) stress was used to analyse the components made from ductile materials, namely the titanium abutment screws and acrylic specimen holders, while the maximum principal stress (σ_1_) and minimum principal stress (σ_3_) were used for components made of zirconia. All values exceeding the ultimate strength limits of the material were presented in the figures with separate top and bottom color bands (purple and grey), indicating the placement and size of these regions. In addition, the Ansys stress tool was used to calculate and plot the safety factor for all zirconia parts according to the Coulomb-Mohr failure criterion. This criterion is typically used to estimate nearness to failure for brittle materials, and the safety factor (FoS) for a specific point in the material is calculated by the tool as:

3 $$FoS= \frac{\sigma_1}{S_UT} +\frac{\sigma_3}{S_UC} ,$$

where *σ*_*1*_ and *σ*_3_ are the maximum and minimum principal stresses, respectively. *S*_*UT*_ denotes the material ultimate tensile strength limit and *S*_*UC*_ is the material's ultimate compression strength limit (stated with its negative value). Each term in the equation is used only if it has the correct sign; σ_1_ must be positive and σ_3_ negative; otherwise, the invalid term is assumed to be negligible and dropped. Local material failure is typically assumed for values of *FoS* < 1. In the present study, the safety factor provides a simple indicator of stress severity that accounts for the material’s different strengths in compression and in tension, not to predict the failure of the components. 

## Results 

The results report the stress observed in each of the implant–abutment complex components (i.e., implant body, abutment, and abutment screw) across different design models. In the OPI model, the implant body and abutment are integrated into a single unit; therefore, OPI results are reported only for stresses in the implant body section.

Tables 5 and 6 summarize the stresses of the individual components, which will be described in detail below. 

**Table 5:** Numerically largest maximum principal, minimum principal, and equivalent stresses for all models

Overall stresses (MPa)	Implant body		Abutment		Abutment screw			Implant -abutment complex	
Design Models	σ_1_	σ_3_	σ_1_	σ_3_	E	σ_1_	σ_3_	E to specimen holder	Deformation (mm)
Model 1 OPI	1737^**†**^	−2135^**†**^	-	-	-	-	-	122	0.19
Model 2 BLTS	1292^**†**^	−1826	1176	−3070^†^*	506	-	-	320	0.39
Model 3 BLCS	1255^**†**^	−1893	> 4000^†^* / 1176	< −4000^†^*/ −2977^†^*	-	> 4000^†^*	<−4000^†^*	295	0.38
Model 4 TLTS	1169	−1735	898	−2731^†^*	613	-	-	269	0.28
Model 5 TLCS	1160	−1598	> 4000^†^*/ 879	< −4000^†^*/ −2562^†^*	-	> 4000^†^*	< −4000^†^*	250	0.27

The numerically largest maximum principal stress (σ_1_), minimum principal stress (σ_3_), and equivalent (von Mises) stress (E) observed in the implant body, abutment, abutment screw, and specimen holder for all models when subjected to the specified 400 N external oblique loading and boundary conditions. Values marked with † are noted to exceed the material strength, while values marked with * indicate that the stress exceeded the material strength by more than 500 MPa. When two stress values are reported, the second value indicates the next highest stress value. The maximum total deformation of each model is listed in the last column. Ultimate strengths for materials are as follows: for zirconia, 1200 MPa tensile and 2000 MPa compressive; for titanium, 1054 MPa

**Table 6:** The locations of the highest maximum principal, minimum principal, and equivalent stresses in all models

Area of maximum stress	Implant body		Abutment		Abutment screw		
Design Models	σ_1_	σ_3_	σ_1_	σ_3_	E	σ_1_	σ_3_
Model 1 OPI	External threads, tensile side, just above the specimen holder^**†**^	External threads, compressive side, just above the specimen holder^**†**^	--				
Model 2 BLTS	External threads tensile side, just above the specimen holder level^**†**^	The internal surface of the implant body, at the level of the implant platform (the implant–abutment interface)	The abutment neck, tensile side	Abutment neck, compressive side, level of implant platform (the implant–abutment interface)^†^*	The top threads of the screw, followed by the tensile side of screw neck	--	--
Model 3 BLCS	External threads tensile side, just above the specimen holder level^**†**^	The internal surface of the implant body, at the level of the implant platform (the implant–abutment interface)	The screw–abutment interface^†^*, followed by the abutment neck, tensile side	The screw–abutment interface^†^*, followed by the abutment neck, compressive side, level of implant platform (the implant–abutment interface)^†^*	--	The screw neck, contact line with abutment (the screw–abutment interface)^†^*	
Model 4 TLTS	External threads tensile side, just above the specimen holder level	The internal surface of the implant body, at the level of the implant platform (the implant–abutment interface)	The abutment neck, tensile side	Abutment neck, compressive side, level of implant platform (the implant–abutment interface)^†^*	The top threads of the screw, followed by the tensile side of screw neck	--	--
Model 5 TLCS	External threads tensile side, just above the specimen holder level	The internal surface of the implant body, at the level of the implant platform (the implant–abutment interface)	The screw–abutment interface^†^*, followed by the abutment neck, tensile side	The screw–abutment interface^†^*, followed by the abutment neck, compressive side, level of implant platform (the implant–abutment interface)^†^*	--	The screw neck, contact line with abutment (the screw–abutment interface)^†^*	

The locations of high maximum principal stress (σ_1_), minimum principal stress (σ_3_), and equivalent (von Mises) stress (E) observed in the implant body, abutment, and abutment screw for all models under the specified 400 N external oblique loading and boundary conditions. Locations marked with † are noted to stress exceeded the material strength, while locations marked with * indicate that the stress exceeded the material strength by more than 500 MPa

For simplicity, one side of the implant body and the abutment was designated as the tensile side and the other as the compressive side, see Fig. 1. This refers to that in present study, the oblique loading geometry induces, in all models, the maximum principal stress (σ_1_) on the side towards which the oblique load is angled (tensile side), and conversely minimum principal stress (σ_3_) on the opposite side (compressive side).

### Stress concentrations in the implant body 

#### Model 1: OPI

The regions of high maximum and minimum principal stresses in the OPI were in the implant body, residing inside the threads on opposite sides of the implant body and positioned slightly above the top surface of the specimen holder. The largest value of the maximum principal stress (σ_1_) recorded was 1737 MPa, and the numerically largest value of the minimum principal stress (σ_3_) was −2135 MPa.

**Fig. 3:**
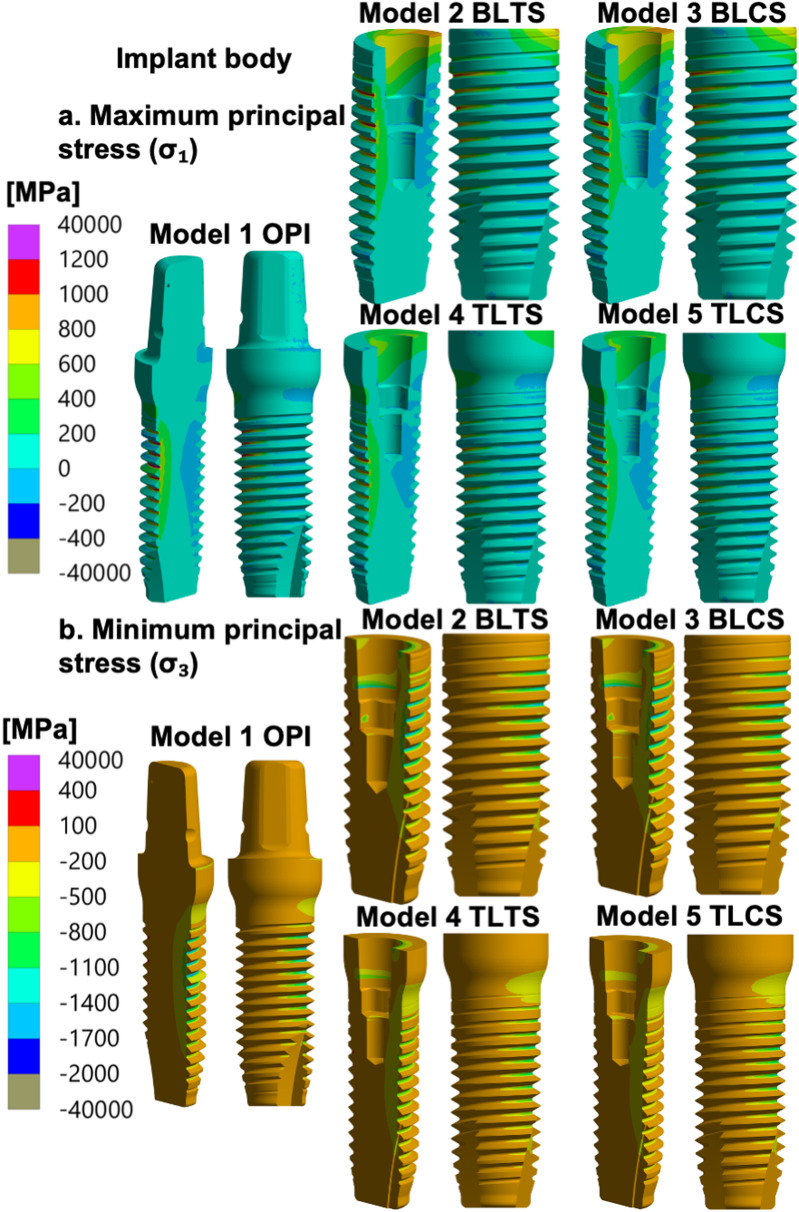
Principal stresses in the implant body of the zirconia implant design models. **a** Maximum principal stress (σ_1_), and (**b**) minimum principal stress (σ_3_) distribution in the implant body of all models: OPI: One-piece implant, BLTS: Bone-level two-piece with a titanium abutment screw, BLCS: Bone-level two-piece with a ceramic abutment screw; TLTS: Tissue-level two-piece with a titanium abutment screw and TLCS: Tissue-level two-piece with a ceramic abutment screw. Stresses (MPa) were calculated for the specified 400 N external oblique loading and boundary conditions

Higher levels of stress were observed in the implant body of the OPI (Model 1) compared to the TPIs (Models 2 to 5). (See Fig. 3a and b and Tables 5 and 6). 

#### Models 2 to 5: TPI models

The maximum and minimum principal stress distributions in the implant body were similar for all TPIs (Models 2 to 5). The largest value of the maximum principal stress (σ_1_) was observed in the external threads on the implant body’s tensile side, located just above the specimen holder. The numerically largest value of the minimum principal stress (σ_3_) was observed at the internal surface of the implant body, at the level of the implant body platform (the implant–abutment interface). 

The TL (Models 4 and 5) showed lower levels of stress when compared to the BL (Models 2 and 3). (See Fig. 3a and b and Tables 5 and 6).

### Stress concentrations in the abutments

#### Models 2 to 5: TPI models

For all abutment models, the largest value of the maximum principal stresses (σ_1_) was observed on the loading side of the abutment neck. Furthermore, all abutments showed large values of minimum principal stress (σ_3_) where the abutment neck was pressed into the implant platform (the implant–abutment interface). 

**Fig. 4:**
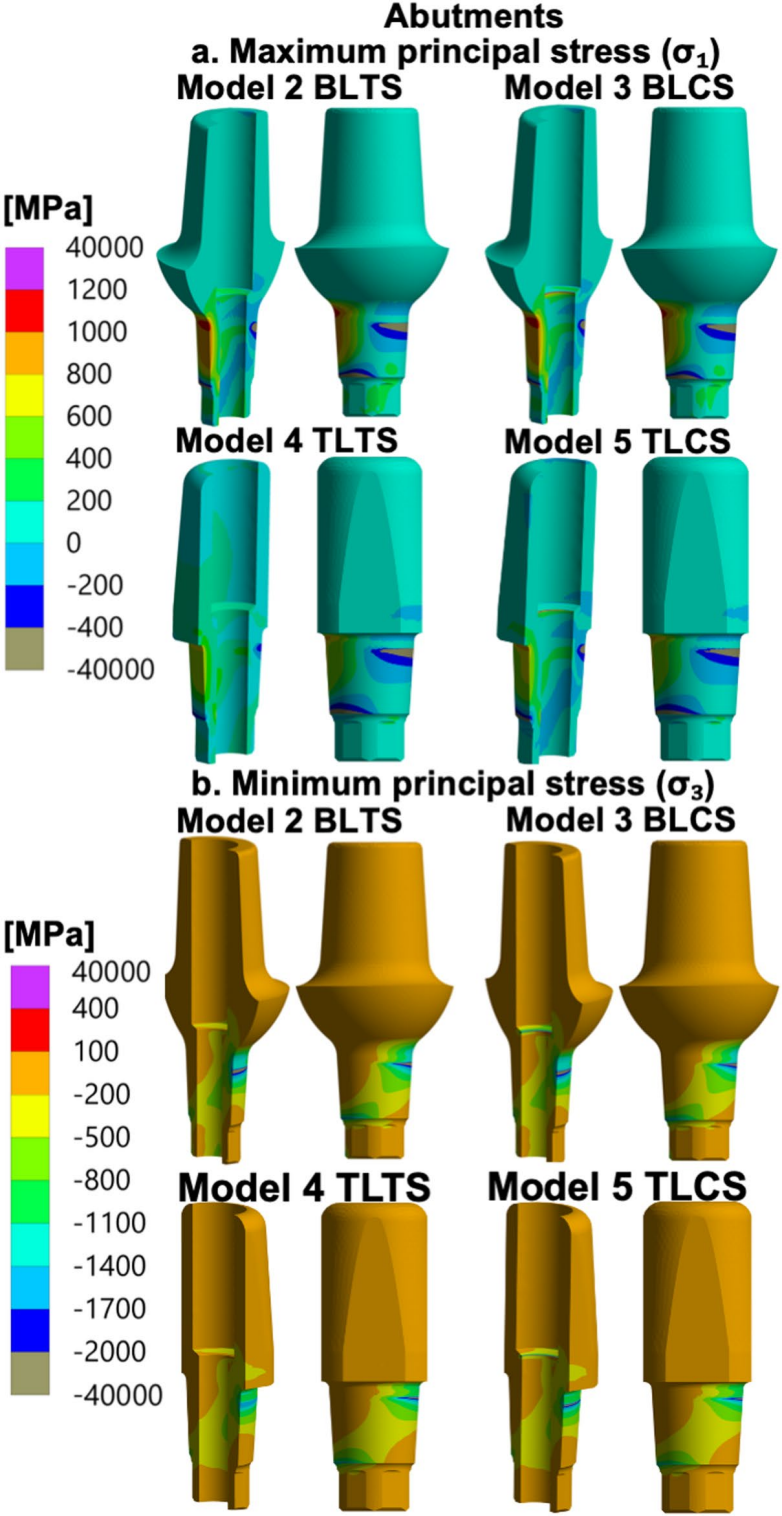
Stresses in the abutments of the zirconia TPIs (Models 2 to 5). **a** Maximum principal stress (σ_1_), and (**b**) Minimum principal stress (σ_3_) distribution in the abutments of Models 2 to 5: BLTS: Bone-level two-piece with a titanium abutment screw, BLCS: Bone-level two-piece with a ceramic abutment screw; TLTS: Tissue-level two-piece with a titanium abutment screw and TLCS: Tissue-level two-piece with a ceramic abutment screw. Stresses (MPa) were calculated for the specified 400 N external oblique loading and boundary conditions

 For the BLCS and TLCS (Models 3 and 5), which have ceramic abutment screws, very high stress values exceeding 4000 MPa were observed in the interface between the screw neck and the abutment (the screw–abutment interface). See Fig. 4a and b and Tables 5 and 6.

### Stress concentrations in the abutment screws

#### Models 2 to 5: TPI models

**Fig. 5:**
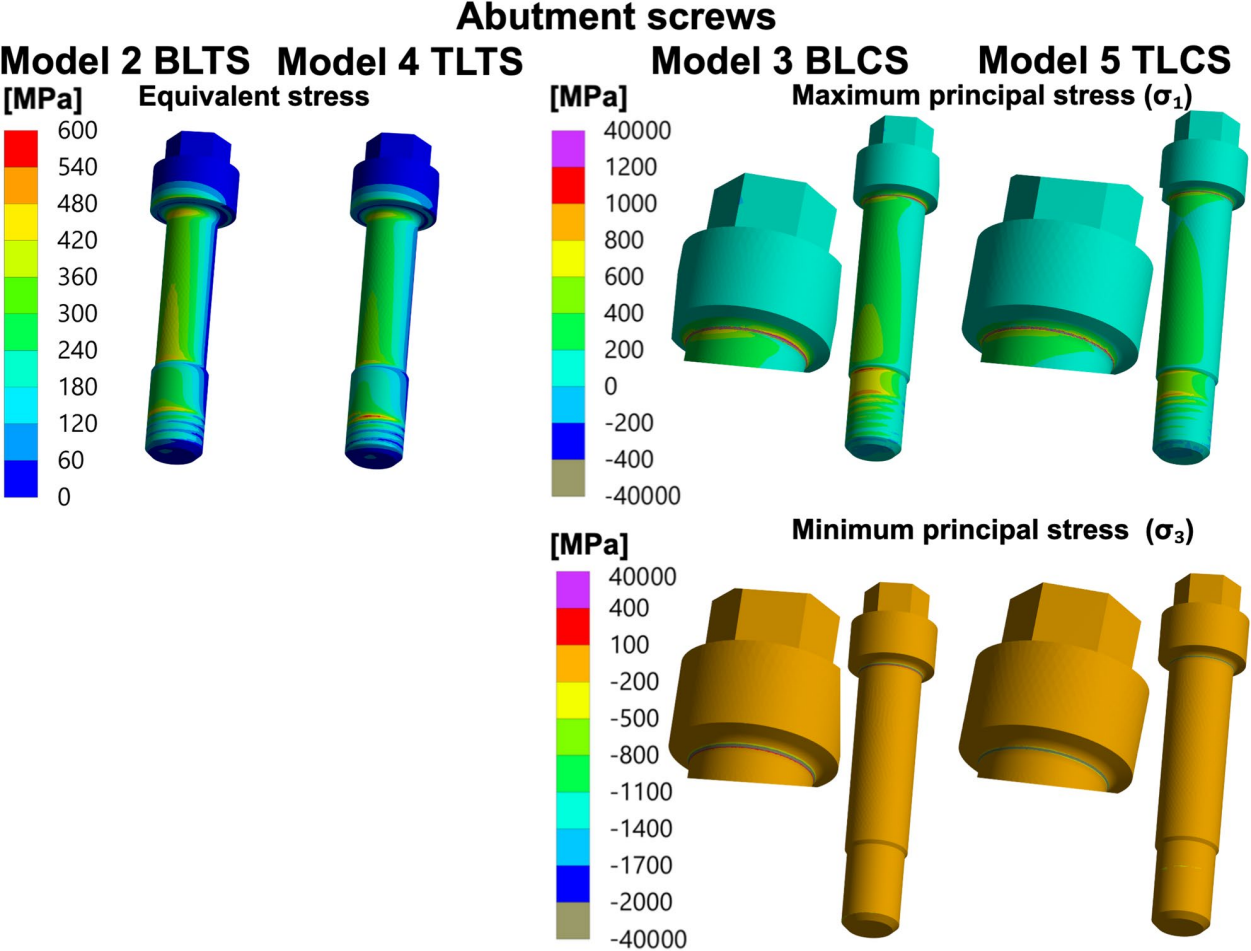
Stresses in the abutment screws of the TPIs (Models 2 to 5). Distribution of the equivalent (von Mises) stress in the titanium abutment screws and the maximum and minimum principal stresses in the ceramic abutment screws for all TPI (Models 2 to 5): BLTS: Bone-level two-piece with a titanium abutment screw, BLCS: Bone-level two-piece with a ceramic abutment screw; TLTS: Tissue-level two-piece with a titanium abutment screw and TLCS: Tissue-level two-piece with a ceramic abutment screw. Stresses (MPa) were calculated for the specified 400 N external oblique loading and boundary conditions

In the BLTS and the TLTS (Models 2 and 4), which use the titanium abutment screws, the maximum equivalent (von Mises) stress was observed on the abutment screws’ top threads, followed by the tensile side of the screws’ neck. In the BLCS and the TLCS (Models 3 and 5), which use the ceramic abutment screw, a very high level of maximum and minimum principal stress values exceeding 4000 MPa were reported in the screw neck, at the contact line with the abutment (the screw–abutment interface). See Fig. 5 and Tables 5 and 6. 

### Stress transferred to the specimen holder

**Fig. 6:**
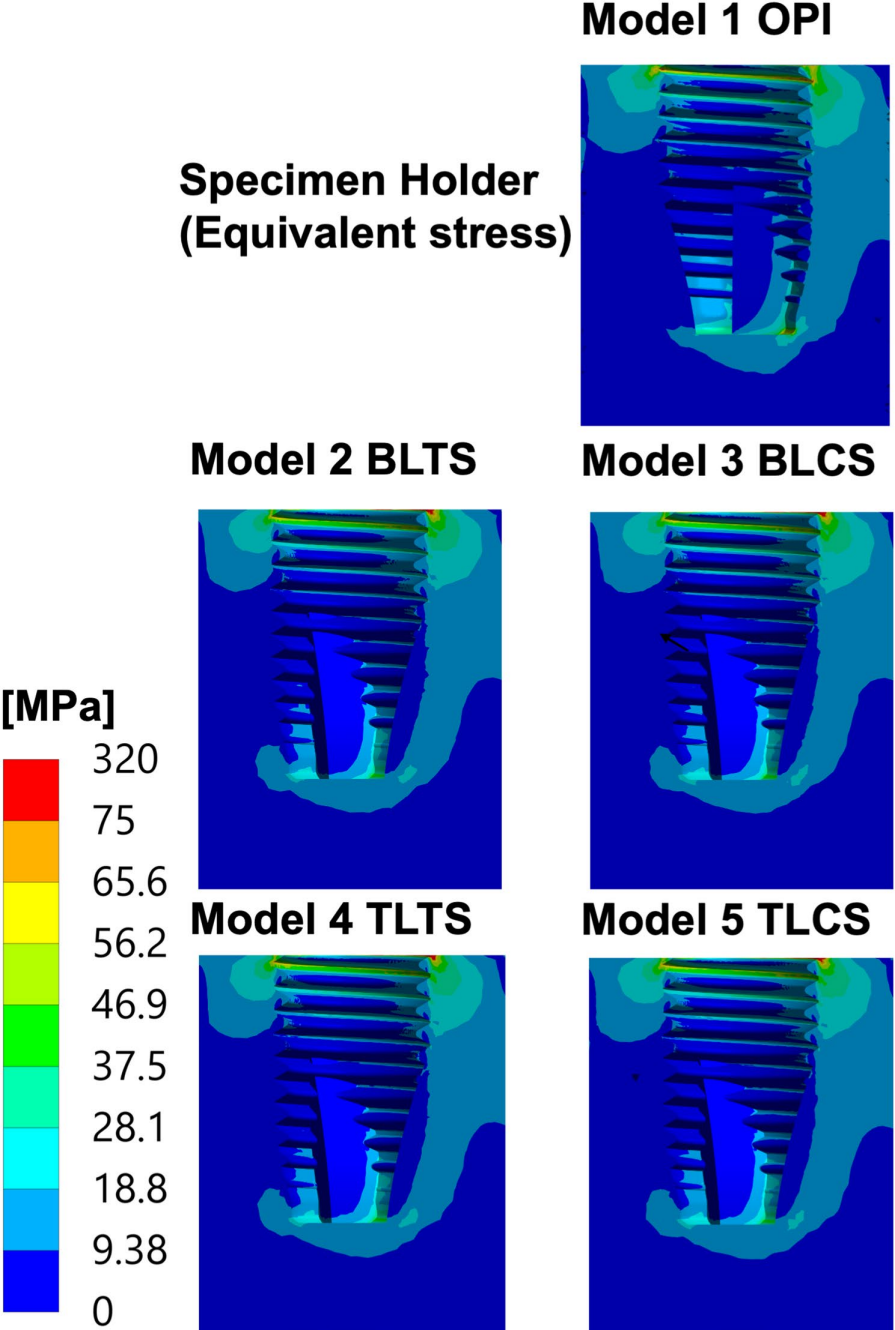
Equivalent (von Mises) stress in the specimen holder of zirconia implant models. The equivalent (von Mises) stress distribution in the specimen holder of all models: OPI: One-piece implant, BLTS: Bone-level two-piece with a titanium abutment screw, BLCS: Bone-level two-piece with a ceramic abutment screw; TLTS: Tissue-level two-piece with a titanium abutment screw and TLCS: Tissue-level two-piece with a ceramic abutment screw. Stresses (MPa) were calculated for the specified 400 N external oblique loading and boundary conditions.

The distribution patterns of the equivalent (von Mises) stress transferred to the specimen holder were similar across all five models. The level of maximum stress in the specimen holder was lower for the OPI compared to all TPI models. A relatively close level of stress was reported among the TPI models, with TL (Models 4 and 5) having slightly lower stress levels than the BL (Models 2 and 3). Additionally, slightly lower stress levels were reported in the models with a ceramic abutment screw (Models 3 and 5) compared to models with a titanium abutment screw (Models 2 and 4). See Fig. 6 and Table 5.

### Total deformation

**Fig. 7:**
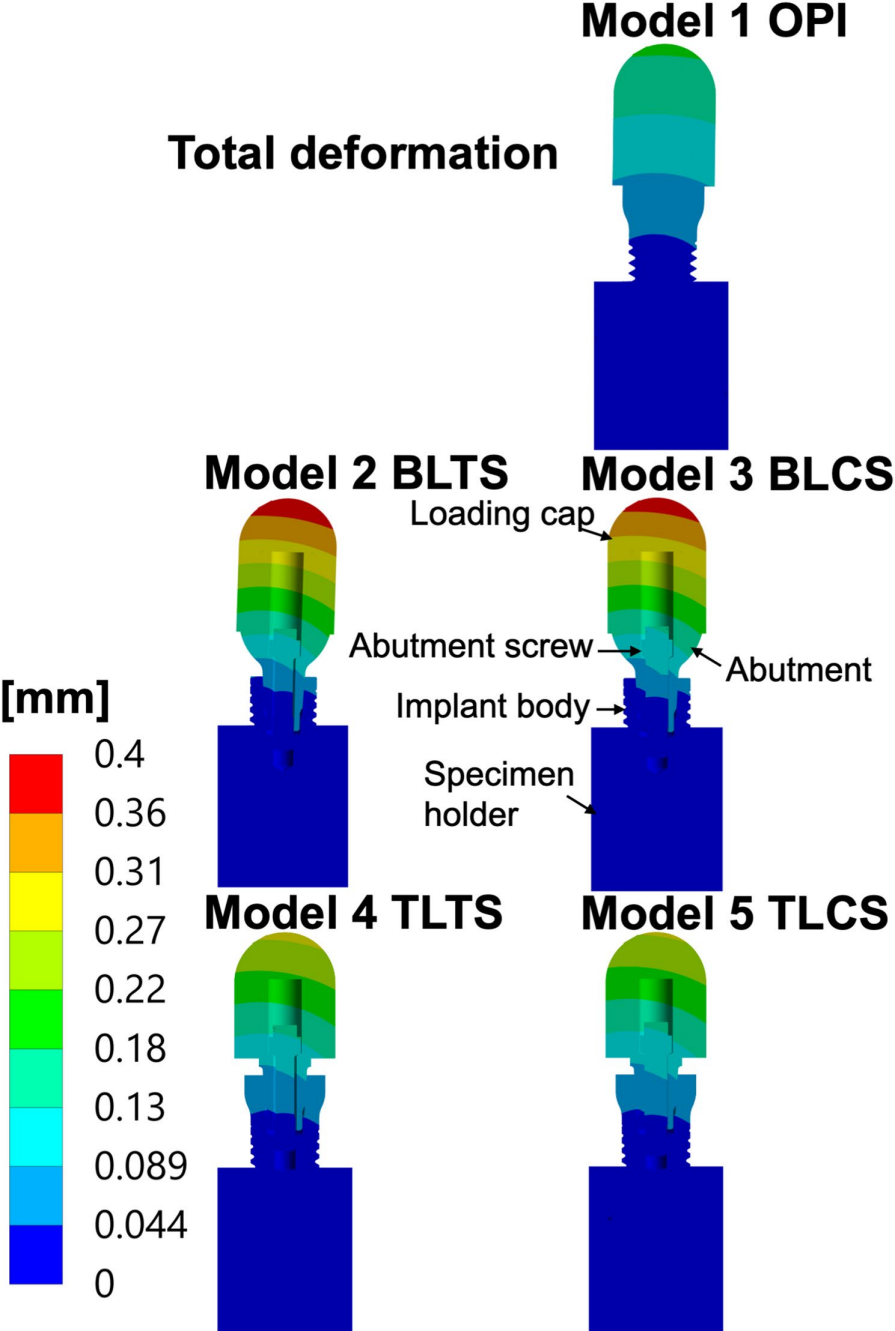
Total deformation (scaled deformation) of all zirconia implant models. The total deformation (scaled deformation) of all models: OPI: One-piece implant, BLTS: Bone-level two-piece with a titanium abutment screw, BLCS: Bone-level two-piece with a ceramic abutment screw; TLTS: Tissue-level two-piece with a titanium abutment screw and TLCS: Tissue-level two-piece with a ceramic abutment screw. The deformation (mm) was calculated for the specified 400 N external oblique loading and boundary conditions

The deformation in the OPI was smaller than in all the TPI (Models 2 to 5). Furthermore, the deformation in the TL models was smaller than that in the BL models, and the deformation of the ceramic abutment screw models was smaller than that of the titanium abutment screw models, which also correlates well with the stress levels in the specimen holder. See Fig. 7 and Table 5. 

### Safety factor

**Fig. 8:**
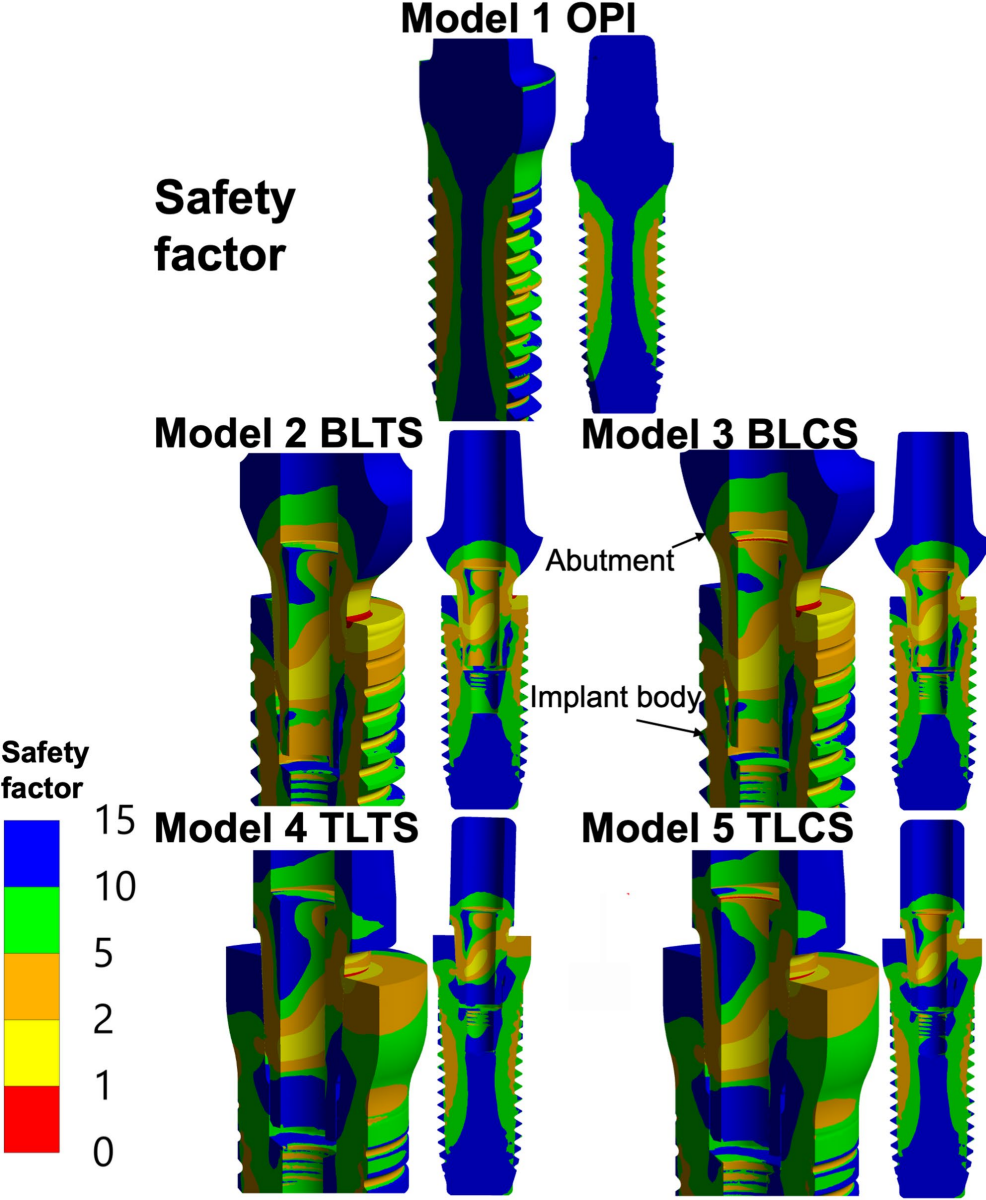
Safety factor was calculated for the specified 400 N external oblique loading and boundary conditions, according to the Coulomb-Mohr criterion. OPI: One-piece implant, BLTS: Bone-level two-piece with a titanium abutment screw, BLCS: Bone-level two-piece with a ceramic abutment screw; TLTS: Tissue-level two-piece with a titanium abutment screw, and TLCS: Tissue-level two-piece with a ceramic abutment screw.

The safety factors (*FoS*) according to the Coulomb-Mohr criterion were calculated, as described in the Methods section, and shown for all five models in Fig. 8. For the OPI, the lowest *FoS* was observed in the external threads. For the TPI models, the lowest *FoS* was found predominantly on the implant platform and abutment neck (implant-abutment interface), both on the compressive side. A localized region of *FoS* < 1 was also observed in the implant body external threads on the tensile side. For the groups using the ceramic screw (BLCS, TLCS), regions of very low *FoS* were observed at the inner edge of the abutment, where the screw neck is in contact (the screw–abutment interface), as well as at the corresponding position on the screw neck. 

## Discussion

The present study investigates zirconia-based BL and TL TPI models. These models incorporate a ceramic abutment screw and are recognized as the first all-ceramic screw-retained TPI systems currently available on the market, comprising an all-ceramic implant body, abutment screw, and abutment [13]. Additionally, the study evaluates the BL and TL TPI with a titanium abutment screw, as well as the OPI zirconia dental implant design models. 

Zirconia is a brittle material that typically fails under normal (tensile) stress; Furthermore, the strength of zirconia varies in tensile and compressive states, necessitating a differentiation between these states. Therefore, the maximum and minimum principal stresses were used in this study to analyse zirconia components, whereas the equivalent (von Mises) stress was used to analyse ductile titanium and acrylic components. [20, 22, 27]. The results of this FEA showed that across all implant-abutment complex models (Models 1 to 5), the maximum principal stress (σ_1_) was highest on the side on which the oblique load was applied, whereas the minimum principal stress (σ_3_) was highest on the opposite side. This occurrence is related to the 30-degree angle (oblique) used in this study at which the load was applied. The oblique load introduces a bending moment in addition to axial compression, resulting in maximum principal stress (σ_1_) on the side towards which the oblique load is applied (tensile side) and conversely, minimum principal stress (σ_3_) on the opposite side (compressive side) [8]. 

For the OPI design (Model 1), the highest stress concentrations were localized in the implant body. Compared with the stress concentrations in the implant body of the other simulated models (Models 2 to 5), the OPI model exhibited the highest stress. Although direct comparison between the OPI and TPI designs may be complicated due to differences based on the design geometry used in this study, the comparison focused on the implant-abutment complex to evaluate stresses in each design. In the OPI, the maximum stress was localized between the threads, slightly above the upper level of the specimen holder. The identified area of maximum stress concentration in the implant body of the OPI coincides with the fracture site reported in a study investigating clinically fractured zirconia-based OPI [19]. Few previous studies have investigated zirconia-based OPI designs in FEA simulation models, they reported similar findings. Cheng et al [28] reported a similar maximum stress area in zirconia-based OPI under a dynamic loading test with a load of 300 N applied at 30 degrees. A similar maximum stress area was also reported under oblique loading by Çağlar et al [7]. This stress distribution can be attributed to the geometric and structural characteristics of the OPI, which is a single-piece without an abutment interface, with a homogeneous and rigid design. Therefore, stresses are more localized in the implant body than in TPI designs, where the load is more distributed throughout the abutment, the abutment screw, and the implant body. 

Other studies evaluated stress concentration in the surrounding bone and reported less stress transferred to the surrounding preimplant area with zirconia-based OPIs compared to zirconia-based TPIs and compared to a titanium-based TPI [8, 29]. This is consistent with the results of the present study, where the zirconia OPI design showed the lowest level of stresses transferred to the specimen holder, although the simplified specimen holder design did not take differences in bone quality into consideration. 

Concerning the implant–abutment complex (including implant body, abutment, and abutment screw), the simulation showed that the OPI model had the lowest level of overall stress concentrations, total deformation, and stresses transferred to the specimen holder compared to the TPI models. 

In the TPI designs, the areas of high maximum principal stress (σ_1_) in the implant body were localized between the implant body external thread above the specimen holder. The areas of highest minimum principal stress (σ_3_) were localized on the internal surface of the implant body, at the level of the implant platform (the implant–abutment interface), where the implant body is pressed by the abutment under loading and due to the tight fit provided by the internal conical connection. A study analysing clinically failed zirconia dental implants reported fractures in the implant body between the threads and in the implant–abutment interface of TPI designs [19]. This matches the area with the high maximum and minimum principal stresses identified in the present study. 

For the abutment and the screw of TPI models, the results were dependent on whether a titanium or ceramic abutment screw design was used. In the case of titanium abutment screw design models (Models 2 and 4), the high minimum principal stress (σ_3_) was localized in the abutment neck, where it is pressed into the implant platform (the implant–abutment interface). In an *in vitro* study following the ISO14801:2016 on TPI designs with a titanium abutment screw, fracture was reported in both the abutment neck at the interface with the implant and the implant platform, corresponding to the area reported to have high stress in the present study [30]. These findings indicate a spatial correspondence between stress concentration regions identified under quasi-static loading and fracture locations reported in experimental and clinical studies, without implying failure prediction. Moreover, a previous FEA study used a BL zirconia-based implant model with a titanium abutment screw and reported consistent areas of high stress concentrations in the implant body, the abutment, and the abutment screw, with the highest equivalent stresses reported in the abutment under oblique loading [25]. Although the designs of these parts were different from those used in the present study, the outcomes are qualitatively consistent with the high minimum principal stress (σ_3_) areas of the TPI with titanium abutment screw models.

With regards to the deformation and the stresses transferred to the specimen holder, the TPI with the titanium abutment screw models reported slightly higher deformation and higher stresses compared to the ceramic abutment screw models (Models 3 and 5). This can be attributed to the ductility of titanium or to the different geometry of the titanium abutment screw, which allows slightly greater elastic accommodation within the model, resulting in increased deformation and higher stresses transferred to the specimen holder. 

**Fig. 9:**
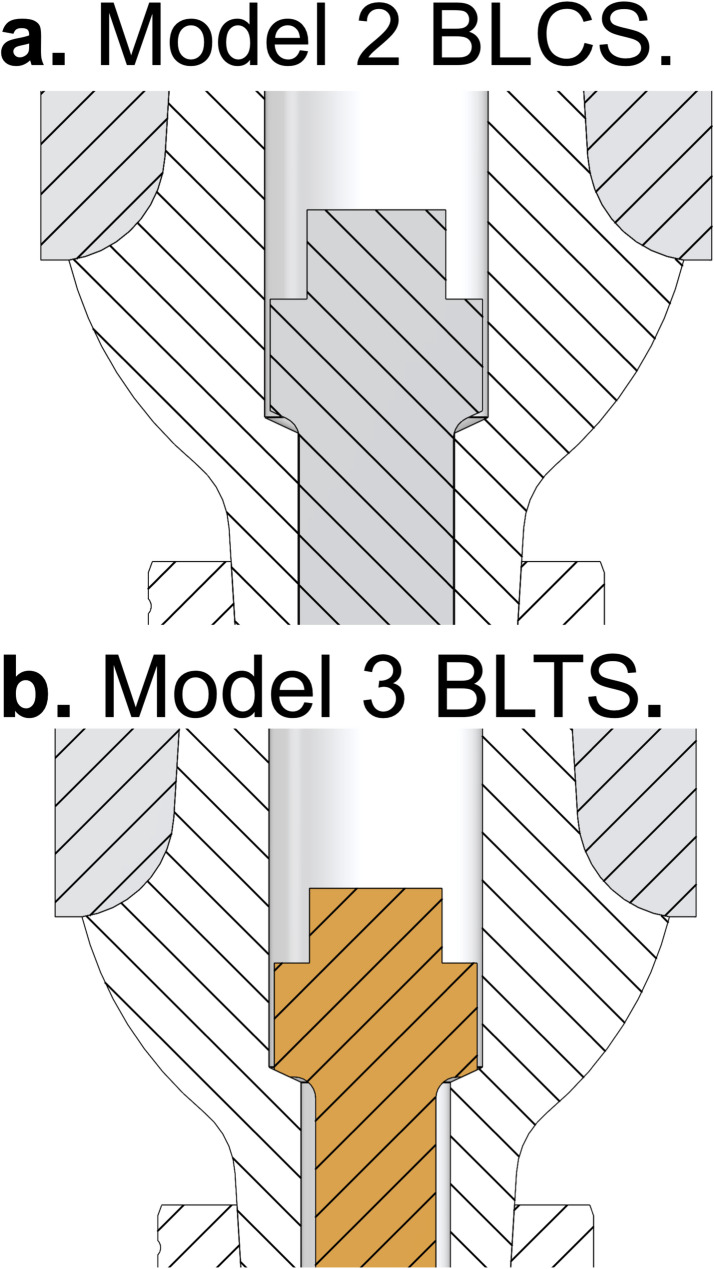
The screw-abutment interface of ceramic and titanium abutment screw designs. This figure aids in visualizing the design geometry of the screw–abutment interface. (a) Shows the screw-abutment connection in the model with a ceramic abutment screw (Model 2); the design has the screw neck seated directly on the relatively sharp inner edge of the abutment screw channel opening (line contact), resulting in high levels of localized stress. (b) Shows the screw to abutment connection for the model using the titanium abutment screw design (Model 4); the screw and abutment have matching conical surfaces with equal opening angles, resulting in the screw resting on a larger contact area and more uniform stress distribution.

For the TPI designs with a ceramic abutment screw (Models 3 and 5), areas of very high localised stress concentration were observed in the screw–abutment interface area. This is due to the ceramic abutment screw design, which has the screw neck seated directly on the relatively sharp inner edge of the abutment screw channel opening, leading to local regions of very high stress in both the abutment and the abutment screw. In contrast, the titanium abutment screw design features a conical surface with equal opening angles, allowing the screw to rest on a larger area and thus reducing local stress concentration. Furthermore, the stress values at the ceramic screw–abutment interface should be interpreted with caution, as this sharp contact geometry is naturally prone to creating singularities and can artificially elevate stress values in FEA simulations, largely due to the use of a linear elastic material model without a damage model. Fig. 9a and b compare the ceramic and titanium abutment screw designs. 

The titanium alloy is frequently considered biomechanically beneficial due to its ductility and capacity to absorb strain energy. However, in the present study, since a titanium-based implant body model was not included, a direct comparison cannot be made to the zirconia-based implant body. Moreover, a direct material comparison of titanium abutment screw with ceramic abutment screws is not possible because their design geometries differ. As a result, the stress and deformation patterns observed are influenced by design features rather than by material properties.

In clinical studies that used zirconia-based TPI designs, abutment fracture was a common technical complication [15-18]. This observation is consistent with the stress pattern identified in the results of this study, which showed elevated minimum principal stress (σ_3_)stresses in the abutment across all TPI design models. Notably, these studies employed different designs and brands compared to those used in the present study models.

A recent *in vitro* study, which followed the ISO 14801:2016 recommendations, compared BL zirconia implants with titanium and ceramic abutment screws, using designs similar to those in the present study (Models 2 and 3). It found comparable fracture resistance values between different abutment screw types in the artificial loading and hydrothermal aging test, indicating that both designs can withstand physiologically relevant normal occlusal loading conditions and possess sufficient fracture resistance and stability for clinical use [13]. In the context of the TL and BL TPIs, the TL (Models 4 and 5) demonstrated lower stress levels in the implant, the abutment, and the specimen holder, as well as reduced minimum principal stress (σ_3_) in the screw and less total deformation. These outcomes may be related to the TL models; compared to the BL (Models 2 and 3), they have a larger surface area and greater zirconia thickness due to the extra tissue collar, which provides additional support in the implant platform area, where the abutment compresses it under loading. This may reduce mobility in the overall system, resulting in less deformation and stress in the implant body and the specimen holder. To date, no previous FEA studies have directly compared zirconia TL and BL dental implant designs. However, existing research involving titanium dental implants has shown that the TL design exhibits lower stress levels compared to the BL design [31, 32]. 

In the results of the safety factor, as shown in Figure 8, the material strength was exceeded at a few stress concentration areas, where the safety factor is < 1, indicating a location where a possible crack initiation may start to form under the specific loading scenario. In the absence of a damage or crack-formation model, this, however, remains just an indication that this is an area of interest. 

Within the framework of the FEA, the zirconia material parts in the models exhibited high local values of both maximum and minimum principal stress. In the process of building the simulation models, all materials were assumed to be linear elastic and isotropic. This assumption is generally appropriate for the titanium screws, whereas for the ceramic screws, it represents a simplified approximation that may contribute to discrepancies between numerical simulations and experimental observations. In addition to the limitations of the material model, this FEA study used only one simulated loading scenario, which assessed performance under very specific conditions and did not include cyclic loading or a bone model. Therefore, it was unsuitable for estimating fatigue life or failure, and a more advanced material model should be developed and tested to capture (at a macro level) the effect of local damage in zirconia. Nevertheless, the linear elastic and isotropic material model was accepted as sufficient since the aim of the analysis was to identify high stress concentration within the investigated design models.

In addition to simplifying assumptions, the produced physical parts may also differ from the CAD models due to production choices and tolerance variations. Further, the loading conditions in a clinical setting or during physiological mastication may differ from the ISO 14801:2016-based geometric and boundary conditions applied in this quasi-static loading condition used in this simulation [33, 34]. Although ISO 14801:2016 was initially created for experimental fatigue tests, its design and loading setup are often used in finite element analysis to simulate a standard worst-case scenario for dental implants [24, 35]. In addition, when used in finite element simulations under quasi-static conditions, it offers a standardized and reproducible framework that supports consistent stress localization, enables comparison across different implant designs, and allows meaningful comparisons with future numerical studies—without implying failure prediction. Additionally, the ISO 14801:2016 loading geometry is also widely used in laboratory experiments, allowing the FEA results to be compared to results from experimental tests.

Hence, future laboratory and clinical studies should be conducted to validate the simulations' findings in the present study. 

## Conclusions

Within the limitations of this study, under a static oblique load applied within the ISO 14801:2016 geometry condition, stress distributions differ between OPI and TPI. TPIs show lower stress within the implant body but higher stress transfer to the specimen holder compared with OPI. Tissue-level TPIs exhibit lower stress than bone-level designs, whereas stresses in the abutment and screw depend on screw design and material. TPIs with ceramic screws exhibit higher stress levels compared to titanium screw. 

## Data Availability

The datasets used and/or analysed during the current study are available from the corresponding author on reasonable request.

## Abbreviations

BL: Bone-level
BLCS: Bone-level two-piece with a ceramic abutment screw
BLTS: Bone-level two-piece with a titanium abutment screw
CAD: Computer-aided design
E: Equivalent (von Mises) stress
ELI: Extra low interstitial
FEA: Finite element analysis
FKN: Normal stiffness factor
HIP: Heat isostatic pressure
µ: Friction coefficient
OPI: One-piece implant
TL: Tissue-level
TLTS: Tissue-level two-piece with a titanium abutment screw
TLCS: Tissue-level two-piece with a ceramic abutment screw
TPI: Two-piece implant
Y-TZP: Yttria-stabilized tetragonal zirconia polycrystal
ZrO2: Zirconia Zirconium dioxide
